# Some Conditions Simulating Renal Calculus

**Published:** 1892-07-23

**Authors:** 


### ON SOME CONDITIONS SIMULATING RENAL CALCULUS.—I.

A patient begins to pass pus in his urine, to suffer from constant lumbar pain on one side, with attacks of typical renal colic, and to pass blood in the urine occasionally. We naturally think at once of renal calculus—these symptoms we know are generally due to renal calculus—but we must not forget that the same symptoms may be produced by other diseases, such as tubercular disease of the kidney. Cases are every now and then being operated on for renal stone which turn out to be renal tubercle, but fortunately such cases are rare. The diagnosis between the two conditions is often a matter of the utmost difficulty, and often they cannot be distinguished. But at any rate we ought to pay the greatest attention to their differential diagnosis, and it is for this reason that the present article is written.

There are two forms of renal tuberculosis. In one the disease begins in the kidney, a tuberculous focus breaks down, an abscess, or abscesses, form and open into the pelvis, the discharge of the contents of the abscess being often accompanied by some hæmaturia. In the other the disease begins in the mucous membrane of the pelvis, and secondarily invades the kidney just as the disease, beginning in the renal cortex, secondarily invades the pelvic mucous membrane. These forms cannot be distinguished satisfactorily clinically, and we do not see any great object in trying to differentiate between them. When the primary disease is in the mucous membrane the hæmaturia is more likely to be slight and intermittent. When in the kidney tissue there may be profuse hæmorrhage when the abscess opens into the pelvis.

Renal tubercle (and, of course, here we do not write of general tuberculosis where numbers of tubercles occur in the kidneys), unfortunately, is very liable to attack both organs. It often is associated with tuberculosis of other parts of the genito-urinary tract, and hence in its diagnosis an examination of the prostate and the vesiculi seminales may be of use.

But the two points of importance in the differential diagnosis given by Hurry Fenwick * are, first, "the appearance of pus in the urine very soon after if not coincidently with the renal pain"; and, secondly, "the powerlessness of absolute rest to affect or subdue the symptoms." He also mentions the family history as a guide, but it is much inferior evidently to these indications. In renal calculus the stone causes pain from its presence before its irritation is sufficient to set up suppuration enough to cause a deposit of pus in the urine, but in tuberculosis the disease may lead to suppuration before tension is enough to cause pain. Again, in stone in the kidney movement, especially jerking movements, such as jumping or riding in a cart, will jerk the stone about and cause irritation in very sensitive renal structures, and thus cause considerable pain. There is nothing in tubercle to be aggravated by movement of this special kind.

In any case in which we are in doubt between renal stone and renal tubercle, we should certainly examine the urine for tubercle bacilli, but in urine they are only found with difficulty. We have seen an erroneous diagnosis of renal tubercle made on the presence of bacilli, which were very like tubercle bacilli, but did not take the stain quite in the characteristic way.

After a time in a kidney affected with renal tubercle the ureter may get blocked by caseous deposit, and then the pelvis becomes distended with pus, and can be felt as a round tense, tender swelling. It may burst in the loin, or open into the colon.

Often the diagnosis can only be made by an exploratory nephrotomy. If no stone is found and the case is one of tubercle, we may do much good by this operation. As Fenwick says: "The lower ureter would be placed at rest, and the stream of irritating urine would be diverted. By splitting the capsule the intraglandular tension would be reduced, and the pain consequent on the backward pressure would be subdued."

Mr. Henry Morris, in his lectures on "The Surgery of the Kidney," published in the *British Medical Journal* of this spring (April 30th), relates the case of a young man who for a year had been suffering from pain in one renal region, with hæmaturia, and passing pus in his urine. Renal calculus was diagnosed by one surgeon, and the question was only finally decided by an exploratory nephrotomy. However, the kidney was found so diseased it was removed. But removal of the kidney or nephrotomy is not recommended as the best treatment in cases of tubercular disease, as so often the other kidney suffers also. Possibly at the time of the operation the affection of the other kidney might not have given rise to symptoms, and yet the disease might have begun and developed later on. When both organs are extensively diseased vomiting is a frequent symptom.

There is another condition to which we wish now to refer, which sometimes simulates renal calculus—we allude to renal malignant growths. In their earlier stage, before they form palpable tumours, they often cause symptoms closely resembling renal calculus. The lumbar pain, the often profuse hæmaturia, the renal colic when the ureter gets blocked by a clot, are all also prominent symptoms of stone. The pain and hæmaturia are, however, like the same symptoms in renal tubercle, and, unlike stone, not increased by exercise. But this is not always so. We have lately had under observation a patient in whom hæmaturia due to cancer of the kidney was distinctly increased by exercise. He had typical renal colic at times, and his kidney was explored, but no stone was found. After the operation a tumour formed, which was palpable through the abdominal wall, and at the post mortem, some months after, the organ was found greatly enlarged by carcinomatous growth. It certainly contained two calculi in one greatly dilated calyx, but they were black soft concretions which could be broken down on the slightest pressure of the finger-nail, and were quite small—both put together not larger than a pea—and evidently formed from the blood-clot in which they were lying.

Even when renal carcinoma or sarcoma forms a swelling which can be felt, it is not always possible to distinguish this swelling from a pyelitis. It may be just as smooth (as it was in the case just referred to), but would not be so distinctly tender, nor would there be a purulent deposit in the urine, increased by the occasional emptying of the distended pelvis, but if the ureter were completely blocked by a calculus, neither would there be any purulent discharge in a case of calculus pyelitis.

*(To be continued.)*

* *British Medical Journal*, May 28th, 1892.

